# Surgical outcomes for lumbar spinal canal stenosis with coexisting cervical stenosis (tandem spinal stenosis): a retrospective analysis of 565 cases

**DOI:** 10.1186/s13018-018-0765-6

**Published:** 2018-03-20

**Authors:** Tsuyoshi Yamada, Toshitaka Yoshii, Naoki Yamamoto, Takashi Hirai, Hiroyuki Inose, Atsushi Okawa

**Affiliations:** Department of Orthopaedic Surgery, Graduate School, Tokyo Medical and Dental University, 1-5-45 Yushima, Bunkyo-ku, Tokyo, 113-8510 Japan

**Keywords:** Tandem spinal stenosis, Lumbar spinal canal stenosis, Spine surgery

## Abstract

**Background:**

Concurrent cervical and lumbar spinal canal stenosis is known as tandem spinal stenosis (TSS). As research on TSS is limited, there is no consensus on the optimal surgical approach to this problem. We evaluated the prevalence and clinical characteristics of TSS in patients with symptomatic lumbar spinal canal stenosis (LCS).

**Methods:**

The authors performed a retrospective analysis of the outcomes of 565 patients who underwent lumbar surgeries performed for symptomatic LCS. In all the patients, both the cervical and lumbar regions were evaluated preoperatively, and we compared TSS patients and non-TSS patients in terms of multiple clinical parameters. In the TSS patients, we investigated the ratio and clinical outcomes of additional cervical surgeries performed on TSS patients.

**Results:**

Two hundred two cases (35.8%) were considered to be TSS. Twenty-eight patients (5.0%) underwent a cervical operation during the follow-up period. There were no differences between the radiographic TSS patients and non-TSS patients in terms of preoperative lumbar-Japanese Orthopedic Association (L-JOA) scores, postoperative L-JOA scores, and the L-JOA recovery rate (14.8 ± 4.4 points vs 14.2 ± 4.6 points, 23.9 ± 4.3 points vs 23.1 ± 4.5 points, 63.7 ± 28.2% vs 60.3 ± 27.9%, respectively), while the TSS group included a greater number of hypertension cases. The recovery rate L-JOA scores of patients who underwent additional cervical surgeries were significantly lower compared with the rate of patients who experienced treatment for only lumbar lesions (62.8 ± 25.8% vs 39.8 ± 35.5%, *p* = 0.0003). However, additional cervical surgery still improved both the cervical myelopathy-Japanese Orthopedic Association (C-JOA) and L-JOA scores in TSS patients with symptomatic cervical lesion (from 10.3 ± 2.8 points to 12.1 ± 3.0 points, *p* = 0.0302; from 14.8 ± 7.3 points to 19.9 ± 5.0 points, *p* = 0.0331, respectively). In these patients, there were no significant differences in the recovery rate of both C-JOA and L-JOA scores between the single-stage surgery group and the staged surgery group (40.7 ± 35.8% vs 20.7 ± 16.1%; 50.9 ± 25.1% vs 34.2 ± 39.3%, respectively).

**Conclusions:**

Radiographic co-existing cervical stenosis did not affect surgical outcomes for LCS, although symptomatic cervical lesion affected neurological score after lumbar surgery. An additional surgery for cervical lesion significantly improved neurological findings in TSS patients.

## Background

Tandem spinal stenosis (TSS) is defined as concurrent cervical and lumbar stenosis. Dagi et al. reported that TSS was characterized by a triad of intermittent neurogenic claudication, progressive gait disturbance, and mixed myelopathy and polyradiculopathy in both the upper and lower extremities [1]. The prevalence of TSS has been reported to range from 5 to 28% [1–3]. When a TSS patient has severe cervical myelopathy, concurrent lumbar spinal canal stenosis (LCS) is often abstrused by symptoms due to a cervical lesion, leading to difficulty in identifying the origin of these neurological findings, especially in the lower limbs. Due to the complicated nature of this condition, research on TSS is limited, and thus, there is no consensus on the optimal surgical approach to this problem [4].

The surgical strategy for patients with TSS is still controversial. Predominantly, symptomatic legions are usually surgically treated. If neurological symptoms originate significantly from both the cervical and lumbar spine, then both lesions require surgical decompression. A staged procedure (cervical followed by lumbar [1, 5] or vice versa [6]) or simultaneous cervical and lumbar decompression for TSS [2, 4, 5, 7] has been advocated. We previously investigated the effectiveness of additional lumbar surgery after cervical decompression for TSS patients who had predominant neurological symptoms originating from the cervical spine and found that the neurological recovery from single-stage surgery was equal to that of staged procedures [8].

In the present study, we investigated symptomatic LCS patients who required lumbar decompression surgery in order to illuminate more details on the epidemiology and pathology of TSS. We also evaluated the effectiveness of additional cervical decompression surgery after lumbar surgery for TSS patients who had predominant clinical symptoms from the lumbar spine.

## Methods

The current retrospective study was approved by our institutional review board. The authors executed a retrospective analysis of the outcomes of 565 elective lumbar surgeries performed in symptomatic LCS patients from July 2010 to July 2016 who had a follow-up of more than 1 year after surgery. There were 286 females and 279 males, with an average age of 70.7 ± 9.5 years (32–92 years).

Clinical symptoms included neurologic claudication and/or radicular pain. In principle, we conducted myelography for all these patients before performing surgeries and evaluated not only the lumbar spine but also the cervical region. The exclusion criteria were cases with tumors of the cauda equina, vertebral compression fractures, single-level disc herniation, isolated lateral or foraminal stenosis, a history of spine surgery, and emergency cases in which myelography was not performed. Medical records of these patients were reviewed for demographics including the length of follow-up, age, gender, habit of smoking, and preoperative co-morbidities such as diabetes mellitus (DM), hypertension (HT), ischemic heart disease (IHD), hemodialysis (HD), collagen disease, obesity, and osteoporosis. The presence of a joint prosthesis (hip or knee) and computed tomography (CT)-detected cervical ossification of the longitudinal ligament (C-OPLL) were also recorded in this study.

Spinal cord and/or cauda equine compressions were evaluated using myelography and CT myelography (CTM). Before the lumbar surgeries, compressive lesions at the cervical levels were also evaluated in addition to the lumbar lesions. After myelography, CT scans were obtained using a multi-slice scanner (Aquilion64, TOSHIBA Medical System Inc., Tokyo) to obtain image data of the cervical and lumbar spine. The scanning parameters were as follows: 120 kV, 100 to 300 mA, 2-mm thickness for slice data, and 2-mm thickness for reconstruction. The scanning time for the cervical and lumbar spine was less than 10 s. No patients displayed neurological deterioration during the CT examinations.

The myelographic criterion for the diagnosis of LCS was narrowing > 50% of the dural sac. The criterion in the cervical spine was an absolute reduction of the dorsoventral dimension of the spinal canal to 10 mm or less and a disruption or block of contrast material flow [1]. In the CTM images, a compressive lesion of the spinal cord or cauda equina was also defined as a lesion in contact with the anterior or posterior aspect of the spinal cord or cauda equina, morphological deformity of the spinal cord, or the disappearance of the subarachnoid space [9]. In addition, ossification of the longitudinal ligament (OPLL) was defined as an ossified mass (≥ 2 mm) arising from the vertebrae [10].

Two independent spinal surgeons evaluated whether TSS was observed or not, and compressive lesions in the whole spine were assessed with a DICOM viewer. The evaluators reached a consensus. Before the image review, the testers read images from the same 60 patients to check the inter-observer agreement. The Kappa coefficient for the inter-observer agreement was 0.71, which was considered to indicate substantial agreement.

The selection of the surgical method was based on multiple factors, such as the location of compressive pathology, the extent of the degenerative process, intervertebral instability, sagittal alignment of the lumbar spine, and medical conditions. In general, the patients got out of bed on postoperative day 2 and started rehabilitation therapies. For external spine stabilization, in the cases treated with cervical fusion, a Philadelphia collar was applied for 3 months. In cases receiving laminoplasty (LAMP), a soft neck collar was used until discharge. In cases treated with a one-level lumbar spine fusion, a soft brace was used postoperatively to immobilize the lumbar spine, but a hard brace was used in cases of multi-level fusions. In these patients, the operative procedure, operation time, blood loss in the cervical operation, and clinical outcomes were investigated. The clinical outcomes were assessed by means of the scoring system proposed by the Japanese Orthopedic Association (JOA); the recovery rate in the cervical case was determined by [(postoperative score − preoperative score)/(17-preoperative score)] × 100%, and the lumbar cases were determined by [(postoperative score − preoperative score)/(29-preoperative score)] × 100% [11].

The demographics of the patients included in this study are shown in Table 1. We compared the non-TSS group with the TSS group in terms of multiple clinical parameters (Table 2). Moreover, we investigated the ratio and clinical outcomes of additional cervical surgery performed on TSS patients.

**Table 1:** Demographics

LCS case, no		565
Disease type	Hypertorophy of the flavum, no	288 (51.0%)
	Disc herniation (> 2 levels), no	60 (10.6%)
	Degenerative kyphosis, no	23 (4.1%)
	Degenerative scoliosis, no	21 (3.7%)
	Spondylolisthesis, no	161 (28.5%)
	L-OYL, no	11 (1.9%)
	L-OPLL, no	1 (0.2%)
Sex, female/male, no		286/279
Age, average (range)		70.7 ± 9.5 (32–92)
YAM, average (range)		90.9 ± 17.0 (48–149)
Surgical method	Decompression/posterior fusion, no	222/343
Follow-up period, average, years		3.2 ± 1.6
Lumbar surgical outcomes	Operative time, average (range), min	220.5 ± 104.0 (44–749)
	Blood loss, average (range), mL	403.9 ± 604.7 (6–6380)
	Preoperative L-JOA score, average (range), points	14.6 ± 4.4 (− 2–24)
	Postoperative L-JOA score, average (range), points	23.6 ± 4.4 (8–29)
	Recovery rate of L-JOA score, average (range), %	62.5 ± 28.1 (− 70.6–100)
	Revisions, no	60 (10.6%)
TSS	Radiological cervical stenosis, no	202 (35.8%)
	Symptomatic cervical stenosis, no	58 (10.3%)
	Cervical operation, no	28 (5.0%)
Preoperative comorbidities	C-OPLL, no	23 (4.1%)
	HT, no	278 (49.2%)
	HL, no	93 (16.5%)
	DM, no	121 (21.4%)
	Smoking, no	159 (28.1%)
	IHD, no	96 (17.0%)
	HD, no	30 (5.3%)
	Collagen disease, no	46 (8.1%)
	Obesity, BMI > 25, no	216 (38.2%)
	Artificial joint, TKA or THA, no	33 (5.8%)
	Osteoporosis, YAM < 70%, no	68 (12.0%)

*Abbreviations*: *LCS* lumbar spinal canal stenosis, *L-OYL* ossification of yellow ligament of the lumbar spine, *L-OPLL* ossification of the posterior longitudinal ligament of the lumbar spine, *YAM* young adult mean, *L-JOA* Japanese Orthopedic Association for lumbar spinal canal stenosis, *TSS* tandem spinal stenosis, *C-OPLL* ossification of the posterior longitudinal ligament of the cervical spine, *HT* hypertension, *HL* hyperlipidemia, *DM* diabetes mellitus, *IHD* ischemic heart disease, *HD* hemodialysis, *BMI* body mass index, *TKA* total knee arthroplasty, *THA* total hip arthroplasty

**Table 2:** Comparison between cases without TSS and those with TSS

	Non-TSS	TSS	*p*
Case, no	363	202	
Sex, female/male, no	190/173	96/106	
Decompression/posterior fusion, no	132/231	90/112	
Age, average (range)	70.0 ± 9.8 (34–88)	72.0 ± 8.8 (32–92)	NS
YAM, average (range)	90.0 ± 16.5 (52–149)	92.5 ± 17.7 (48–137)	NS
Operative time, average (range), min	221.4 ± 101.7 (50–746)	218.8 ± 108.3 (44–749)	NS
Blood loss, average (range), mL	420.7 ± 663.8 (6–998)	372.8 ± 478.1 (6–4150)	NS
Preoperative L-JOA score, average (range), points	14.8 ± 4.4 (0–24)	14.2 ± 4.6 (− 2–24)	NS
Postoperative L-JOA score, average (range), points	23.9 ± 4.3 (8–29)	23.1 ± 4.5 (9–29)	NS
Recovery rate of L-JOA score, average (range), %	63.7 ± 28.2 (− 70.6–100)	60.3 ± 27.9 (− 57.1–100)	NS
Revisions, no	36 (9.9%)	24 (11.9%)	NS
Symptomatic cervical stenosis, no	0 (0%)	58 (28.7%)	
Cervical operation, no	0 (0%)	28 (13.9%)	
C-OPLL, no	0 (0%)	23 (11.3%)	
HT, no	166 (45.7%)	112 (55.4%)*	0.0268
HL, no	59 (16.2%)	34 (16.8%)	NS
DM, no	73 (20.1%)	48 (23.8%)	NS
Smoking, no	96 (26.4%)	63 (31.2%)	NS
IHD, no	61 (16.8%)	35 (17.3%)	NS
HD, no	14 (3.9%)	16 (7.9%)*	0.039
Collagen disease, no	30 (8.3%)	16 (7.9%)	NS
Obesity, BMI > 25, no	133 (36.6%)	83 (41.0%)	NS
Artificial joint, TKA or THA, no	22 (6.1%)	11 (5.4%)	NS
Osteoporosis, YAM < 70%, no	45 (12.4%)	23 (11.4%)	NS

*Abbreviations*: *TSS* tandem spinal stenosis, *YAM* young adult mean, *L-JOA* Japanese Orthopedic Association for lumbar spinal canal stenosis, *C-OPLL* ossification of the posterior longitudinal ligament of the cervical spine, *HT* hypertension, *HL* hyperlipidemia, *DM* diabetes mellitus, *IHD* ischemic heart disease, *HD* hemodialysis, *BMI* body mass index, *TKA* total knee arthroplasty, *THA* total hip arthroplasty, *NS* not significant

*p < .05

Statistical analysis was performed using an unpaired *t* test and one-way ANOVA followed by Tukey-Kramer’s post hoc test and Fisher’s exact test. All data are expressed as the mean ± standard deviation (SD). A *p* value less than 0.05 was considered to indicate a statistically significant difference.

## Results

For the disease types, 565 LCS cases included 161 cases with spondylolisthesis, 11 cases with ossification of yellow ligament of the lumbar spine (L-OYL), and 1 case with lumbar OPLL (L-OPLL), for which we performed decompression in 222 cases and posterior fusion in 343 cases. The preoperative comorbidities included DM in 121 cases, HT in 278 cases, IHD in 96 cases, HD in 30 cases, collagen disease in 46 cases, obesity (body mass index > 25) in 216 cases, and osteoporosis (young-adult mean < 70%) in 68 cases. Thirty-three patients had undergone total knee or hip arthroplasties prior to surgery for cervical lesions. There were 23 cases with CTM-detected C-OPLL (4.1%) (Table 1).

From the radiographic findings, there were 202 cases (35.8%) with radiological cervical spinal cord compression in LCS patients who underwent lumbar surgeries. These patients were included in the TSS group. The patients in this study tolerated the surgical procedure in the lumbar lesion well and were followed up for an average of 3.2 years (1.0–6.0 years). With regard to lumbar surgery, the average operative time was 220.5 ± 104.0 min. The estimated blood loss was 403.9 ± 604.7 g. Revised lumbar surgeries were applied in 60 of the 565 patients (10.6%) due to perioperative complications such an infection, trouble with instrumentation, recurrence of stenosis, and adjacent segment disease (Table 1). Twenty-eight patients (5.0%) actually underwent a cervical operation during the follow-up periods. Eleven patients underwent these procedures simultaneously. Seventeen patients underwent a two-stage surgery for TSS.

Second, in terms of the presence of TSS in LCS patients, there were no significant differences in sex, age, the average operative time, the average intraoperative blood loss, and the incidence of revision cases between the non-TSS group and the TSS group. With regard to clinical outcomes, there were no differences between the non-TSS groups and the TSS groups in terms of preoperative lumbar-Japanese Orthopedic Association (L-JOA) scores, postoperative L-JOA scores, and the L-JOA recovery rate. Substantial recovery of neurologic symptoms was observed in both groups. The TSS group contained a greater number of C-OPLL patients. The group also included a greater number of HT and HD patients. The rate of coexistent symptomatic cervical canal stenosis was up to 28.7% in the TSS group (Table 2).

Whether there was a single-stage simultaneous cervical and lumbar surgery or a staged surgery, we investigated to what extent performing a cervical operation in addition to lumbar surgery could affect the postoperative outcome of TSS patients. We divided these 202 TSS patients into two groups according to the presence of additional operations. The mean period between cervical and lumbar lesion surgeries was 1.0 ± 1.3 years. The mean recovery rate of cervical myelopathy-Japanese Orthopedic Association (C-JOA) scores at the final follow-up point was 28.5 ± 31.3% when the operation for cervical lesions was applied simultaneously or following lumbar surgery. Postoperative L-JOA scores and the recovery rate of L-JOA scores in the patients who underwent additional cervical surgeries were significantly lower compared with the rate in the patients who experienced the treatment for only lumbar lesions (23.5 ± 4.3 points vs 19.9 ± 5.0 points, *p* = 0.0005; 62.8 ± 25.8% vs 39.8 ± 35.5%, *p* = 0.0003, respectively). Nevertheless, in the additional cervical surgery group, the L-JOA score before cervical surgery was 14.8 ± 7.3 points, but it significantly improved to 19.9 ± 5.0 points after cervical surgery (*p* = 0.0331) (Table 3). This group also showed significant improvement in C-JOA scores from 10.3 ± 2.8 points to 12.1 ± 3.0 points (*p* = 0.0302).

**Table 3:** Comparison between cases without cervical surgery and those with cervical surgery

	Only lumbar operation	Additional cervical operation	*p*
Case, no	174	28	
Sex, female/male, o	83/91	13/15	
Decompression/posterior fusion, no	67/107	23/5	
One-stage/two-stage additional operation	–	11/17	
Period between cervical and lumbar surgery, average, years	–	1.0 ± 1.3 (0–5)	
Age, average (range)	72.2 ± 8.5 (32–89)	70.8 ± 10.6 (43–92)	NS
YAM, average (range)	92.4 ± 17.8 (48–137)	93.8 ± 17.4 (69–127)	NS
Operative time, average (range), min	221.4 ± 108.5 (44–749)	201.6 ± 107.4 (70–572)	NS
Blood loss, average (range), mL	381.5 ± 493.1 (6–4150)	315.5 ± 366.5 (70–1450)	NS
Pre lumbar operative L-JOA score, average (range), points	14.3 ± 4.5 (− 2–24)	13.2 ± 5.4 (− 2–22)	NS
Pre cervical operative L-JOA score, average (range), points	–	14.8 ± 7.3 (3–26)	
Postoperative L-JOA score, average (range), points	23.5 ± 4.3 (9–29)*	19.9 ± 5.0 (11–29)	0.0005
Recovery rate of L-JOA score, average (range), %	62.8 ± 25.8 (− 8.3–100)*	39.8 ± 35.5 (− 57.1–100)	0.0003
Preoperative C-JOA score, average (range), points	–	10.3 ± 2.8 (5–15)	
Postoperative C-JOA score, average (range), points	–	12.1 ± 3.0 (6.5–17)	
Recovery rate of C-JOA score, average (range), %	–	28.5 ± 31.3 (0–100)	
Symptomatic cervical stenosis, no	30 (17.2%)	28 (100%)	
Revisions, no	24 (13.8%)	0 (0%)	
C-OPLL, no	17 (9.8%)	6 (21.4%)	NS
HT, no	97 (55.7%)	15 (53.6%)	NS
HL, no	26 (14.9%)	8 (28.6%)	NS
DM, no	40 (23.0%)	8 (29.3%)	NS
Smoking, no	57 (32.8%)	6 (21.4%)	NS
IHD, no	31 (17.8%)	4 (14.3%)	NS
HD, no	12 (6.9%)	4 (14.3%)	NS
Collagen disease, no	15 (8.6%)	1 (3.6%)	NS
Obesity, BMI > 25, no	71 (40.8%)	12 (42.9%)	NS
Artificial joint, TKA or THA, no	10 (5.7%)	1 (3.6%)	NS
Osteoporosis, YAM < 70%, no	23 (13.2%)	0 (0%)	

*Abbreviations*: *YAM* young adult mean, *L-JOA* Japanese Orthopedic Association for lumbar spinal canal stenosis, *C-JOA* Japanese Orthopedic Association for cervical myelopathy, *C-OPLL* ossification of the posterior longitudinal ligament of the cervical spine, *HT* hypertension, *HL* hyperlipidemia, *DM* diabetes mellitus, *IHD* ischemic heart disease, *HD* hemodialysis, *BMI* body mass index, *TKA* total knee arthroplasty, *THA* total hip arthroplasty, *NS* not significant

*p < .05

The present study included 30 patients who had symptomatic cervical lesions but never experienced cervical operations. Their mean recovery rate of L-JOA scores at the final follow-up was 56.4 ± 27.0%. The additional cervical operation group (*n* = 28) could not get over these outcomes (56.4 ± 27.0% vs 39.8 ± 35.5%, NS). Severe cervical lesions, to the extent they needed operations, could be profoundly attributed to poor outcomes after lumbar and cervical surgeries. Further, except for operative times, there were no significant differences in various clinical parameters, even in the recovery rate of both C-JOA and L-JOA scores between the single-stage surgery group and the staged surgery group (Table 4).

**Table 4:** Comparison between cases with single-stage operations and those with two-stage operations

	Single-stage	Two-stage	*p*
Case, no	11	17	
Sex, female/male, no	4/7	9/8	
Decompression/posterior fusion, no	11/0	12/5	
Period between cervical and lumbar surgery, average, years	0	1.7 ± 1.3 (0.2–5)	
Age, average (range)	66.7 ± 10.2 (48–82)	73.4 ± 10.4 (44–85)	NS
YAM, average (range)	97.4 ± 19.6 (69–127)	92.2 ± 16.9 (69–123)	NS
Operative time, average (range), min	257.6 ± 134.1 (117–572)*	168.6 ± 74.2 (70–349)	0.0348
Blood loss, average (range), mL	432.3 ± 486.6 (70–1450)	253.8 ± 282.4 (10–1208)	NS
Pre lumbar operative L-JOA score, average (range), points	14.0 ± 4.5 (8–21)	12.8 ± 5.9 (−2–22)	NS
Pre cervical operative L-JOA score, average (range), points	–	14.8 ± 7.3 (3–26)	
Postoperative L-JOA score, average (range), points	21.7 ± 4.3 (18–29)	19.0 ± 5.2 (11–28)	NS
Recovery rate of L-JOA score, average (range), %	50.9 ± 25.1 (21.4–100)	34.2 ± 39.3 (− 57.1–90.9)	NS
Preoperative C-JOA score, average (range), points	10.5 ± 2.6 (7–13)	10.2 ± 2.9 (5–15)	NS
Postoperative C-JOA score, average (range), points	12.7 ± 3.1 (7–15.5)	11.7 ± 3.0 (6.5–16)	NS
Recovery rate of C-JOA score, average (range), %	40.7 ± 35.8 (0–100)	20.7 ± 16.1 (0–80)	NS

*Abbreviations*: *YAM* young adult mean, *L-JOA* Japanese Orthopedic Association for lumbar spinal canal stenosis, *C-JOA* Japanese Orthopedic Association for cervical myelopathy, *NS* not significant

*p < .05

## Discussion

Because the spondylotic process in the spine may initially present as an isolated segmental problem, there are often multiple levels of concurrent degenerative pathology [12] that are recorded as TSS. The pathology of TSS is complicated, and the interpretation of clinical outcomes seems controversial and difficult. Due to the limited number of these patients, a clearly defined prevalence and surgical algorithm do not exist. The aim of this retrospective study was to determine the prevalence of myelography/CTM-detected cervical canal stenosis that could be either asymptomatic or could manifest itself as a symptomatic cervical lesion in the patients with symptomatic LCS who underwent lumbar surgeries.

Lee et al. reported that magnetic resonance imaging (MRI)-detected moderate and severe cervical cord compression is observed in 24% of LCS patients [13]. From the radiographic findings in the present study, there were 202 TSS cases (35.8%) in 565 LCS patients. The radiological TSS rate was higher compared with the previous report, probably because the LCS patients included in our study had relatively more severe symptoms that required surgery. The rate of coexistent clinically symptomatic cervical canal stenosis was 28.7% in the radiological TSS patients, and 13.9% of the radiological TSS cases needed actual cervical operations. These results seem to support the theory that the presence of symptomatic LCS increases the risk of spondylotic cervical cord compression [14, 15].

Most previous studies have used MRI to evaluate spinal stenosis in the cervical and lumbar spine. However, CTM offers great advantages for visualizing bony compressive lesions. In this study, we used CTM to evaluate the TSS patients because OPLL and ossification of yellow ligament (OYL) are occasionally causative factors for spinal stenosis. In both OPLL cases and spondylotic (non-OPLL) cases, the stenotic lesions, which need surgery, exist at multiple areas in the whole spine. On the other hand, the previous study reported that OPLL cases had a tendency to develop into TSS at a relatively younger age when compared with spondylotic cases [8]. There is also a difference in the distribution of stenotic lesions between the OPLL and spondylotic cases. The incidence of OPLL/OYL in the lower lumbar spine was quite low (2.1% in this study), whereas the spondylotic process was mainly observed in the middle cervical and in the lower lumbar spine [8, 16].

In the evaluation of clinical symptoms, there were no differences in the L-JOA recovery rate between the non-TSS group and the TSS group. When we compared the clinical outcomes of the only lumbar operation group with those of the additional cervical operation group in TSS cases, the latter group showed a lower recovery rate of L-JOA scores, even after both lesions were completely decompressed. From these results, the radiological cervical cord compression itself (radiological TSS) does not always have an effect on clinical outcomes after lumbar surgery. However, in the TSS cases who had symptomatic cervical stenosis that needed surgery, the cervical lesion can affect the lumbar neurological score after lumbar surgeries. Additional cervical decompression can provide some improvements in lumbar symptoms because the lumbar neural fibers may also be under compression in the cervical spondylotic processes [9]. Indeed, 28 additional cervical surgeries for TSS in our study significantly improved not only C-JOA scores but also L-JOA scores from 14.8 ± 7.3 points to 19.9 ± 5.0 points post-cervical decompression. From these findings, we concluded that the prognosis of TSS with symptomatic LCS was no worse than non-TSS, while the prognosis can be significantly influenced by symptoms of the isolated cervical stenosis. However, the additional cervical surgery can still produce some improvement in LCS patients who have symptomatic cervical lesions.

It remains unknown which procedure, two-staged or single-stage surgery, is more effective for achieving postoperative neurologic improvement in TSS patients. When both cervical and lumbar stenoses appeared equally symptomatic, we generally address the cervical spine first or choose a simultaneous procedure. However, in patients included in this study, most cases have severer symptoms in the lumbar spine compared to the cervical spine. Therefore, we performed lumbar surgery first for the patients included in this study. In some limited patients (1.9%), we chose a simultaneous procedure. However, neurological recovery of simultaneously single-stage surgery in TSS was equal to that of staged surgery in the present study. Furthermore, as described above, 28 patients in our study received additional cervical surgery after averaged 1.0 year later than the first lumbar surgery and showed significant neurological recovery not only in cervical neurological scores but also in lumbar scores. These results suggested that cervical operations can be staged if the single-stage surgery is considered to be too invasive for the patient. Eskander et al. reported that age increased the risk of major and minor complications regardless of the surgical algorithm chosen to manage TSS [4]. Our previous study revealed that TSS with myelopathy was associated with age, HT, and DM, whereas TSS with symptomatic LCS was related to HT and HD in the present study. TSS could be related to lifestyle-related disease. Therefore, surgical management should be tailored to the patient’s age and general condition, whether it is by simultaneous or staged procedures.

This study has several other limitations, including the following: (1) the patient baseline was not controlled; (2) this was a retrospective investigation; and (3) this was not population-based. These limitations could be partially ascribed to the challenges of TSS-related surgery. Prospective data collection is needed in the future to clarify more precise clinical outcomes in TSS cases. We must also take the potential risk of radiation by CT scanning into consideration. All patients in this study underwent CTM as a preoperative examination to determine the approach or the vertebral level at which the surgical procedure should be performed.

The subjects in the previous study were limited to TSS cases with myelopathy in which the operations of cervical lesions were prior to lumbar lesions [8]. The present study approached the cases in which lumbar decompressions are prior to cervical lesions to disclose TSS epidemiology and pathology beyond this limitation. The study indicated that the severity of cervical lesions could have a more critical impact on neurological improvement after lumbar surgery for TSS. Given these results combined with our previous data, the authors concluded that TSS could be associated with lifestyle-related disease and that an additional surgery for another lesion significantly improved neurological findings in TSS patients.

## Conclusion

Radiographic co-existing cervical stenosis did not affect surgical outcomes for LCS, although symptomatic cervical lesion affected neurological score after lumbar surgery. An additional surgery for cervical lesion improved neurological findings in TSS.

## Abbreviations

CT: Computed tomography
CTM: CT myelography
DM: Diabetes mellitus
HD: Hemodialysis
HT: Hypertension
IHD: Ischemic heart disease
JOA: The Japanese Orthopedic Association
LAMP: Laminoplasty
LCS: Lumbar spinal canal stenosis
MRI: Magnetic resonance imaging
NS: Not significant
OPLL: Ossification of the longitudinal ligament
OYL: Ossification of yellow ligament
TSS: Tandem spinal stenosis
